# Nano-SPRi Aptasensor for the Detection of Progesterone in Buffer

**DOI:** 10.1038/srep26714

**Published:** 2016-05-24

**Authors:** Effat Zeidan, Renuka Shivaji, Vincent C. Henrich, Marinella G. Sandros

**Affiliations:** 1 Department of Nanoscience, University of North Carolina at Greensboro, Greensboro, NC 27401, USA; 2 Molecular Core Lab, University of North Carolina at Greensboro, Greensboro, NC 27402, USA; 3 Center for Biotechnology, Genomics, and Health Research, University of North Carolina at Greensboro, Greensboro, NC, 27402, USA; 4 HORIBA Scientific, Edison, NJ 08820, USA

## Abstract

Progesterone is a steroid hormone that plays a central role in the female reproductive processes such as ovulation and pregnancy with possible effects on other organs as well. The measurement of progesterone levels in bodily fluids can assist in early pregnancy diagnosis and can provide insight for other reproductive functions. In this work, the detection of progesterone was examined by integrating novel aptamer development with a nanoEnhanced surface plasmon resonance imaging sensor. First, we developed X-aptamers and selected them for binding to progesterone. Then, we took advantage of the multi-array feature of SPRi to develop an optimized biosensor capable of simultaneously screening the 9 X-aptamers developed to determine the binding capabilities of each aptamer. The sensor surface design conditions were further optimized for the sandwich assay, which employed nanoEnhancers (NIR-streptavidin coated quantum dots) for ultrasensitive detection of progesterone molecules. The assay designed was examined over a concentration range of 1.575 ng/mL to 126 μg/mL resulting in a limit of detection (LOD) of 1.575 ng/mL (5 nM) in phosphate buffer.

Surface plasmon resonance imaging (SPRi) is a technology that lies at the forefront in the development of label-free detection and analysis of a variety of biomolecular interactions. DNA, peptides, antibodies, steroids, toxins, and many more biological molecules have been intensively studied in a label-free and real-time manner^1^. This optical assay requires the immobilization of ligands onto a thin metal-coated surface (mostly gold) and utilizes the changes in refractive index at the interface between the metal surface and the medium of interaction as means for the detection (Fig. 1). The size of the analyte studied imposes a limitation on the applicability of this optical sensor, since the sensitivity relies mainly on the mass of the interacting molecule, known as the mass loading effect. This has led to considerable advancement in surface design as well as the use of signal amplification probes to achieve small molecules sensing^2^,^3^,^4^. These latest advancements respond to the need for assessing many biochemical processes of small molecules for clinical and environmental purposes.

Steroid hormones are a family of small hydrophobic analytes derived from cholesterol that have been the targets of many clinical assays from a variety of bodily fluids and at very low concentrations^5^. Progesterone, composed of four cyclic hydrocarbons containing oxygenated functional groups such as ketones, serves as an indicator of early pregnancy, and participates in controlling hormonal levels throughout the reproductive cycle. Its sensitive detection in diverse matrices can assist in fertility investigations^6^,^7^. It normally ranges in level from less than 1 ng/ml in serum during the pre-ovulation phase of the menstrual cycle up to 20 ng/mL in mid cycle and to 300+ ng/mL in pregnancy. Immunoassays such as ELISA^8^, electrophoresis-chemiluminescence detection^9^, and non-competitive idiometric or fluoroimmunoassays^10^, have been the most widely used methods for the detection of progesterone in different matrices. These tests require very carefully controlled procedures, skilled experts to carry out the analysis, and labeled probes to detect the steroid. However, the major drawback of these clinical tests is the high levels of interference arising from the structural similarities among different steroid hormones^5^.

SPRi biosensor technology offers a reliable analytical tool to generate accurate and label-free signal detection of progesterone, real-time data acquisition, and semi-automated operation. This potential platform has been utilized to measure progesterone by employing well-developed immunoassays that utilize monoclonal antibodies as the binding ligands and sensitive surfaces. For example, an inhibition immunoassay procedure was adapted for measuring progesterone levels in cow’s milk and human saliva to achieve a limit of detection of 3.5 ng/mL^11^ and by further varying a number of parameters the same group was able to enhance the sensitivity down to 0.4–0.6 ng/mL in milk and 35–60 pg/mL in HBS-EP buffer^7^. Another study evaluated progesterone conjugated to ovalbumin linkers of varying lengths to determine the effect of these linkers on the anti-progesterone binding activity to achieve a sensitivity range from 0.1–50 ng/mL^12^. Signal enhancement probes such as gold nanoparticles were also investigated for their potential to detect progesterone conjugated to ovalbumin to lower the limit of detection to 4.9 ng/L in an inhibition immunoassay^13^. A similar platform was developed by Mitchell *et al.*, which consisted of immobilizing progesterone on the surface with oligoethylene glycol linkers and gold-streptavidin labels to lower the detection limit to 8.6 pg/mL^14^. As concluded from these studies, the SPR systems are capable of providing means to detect progesterone. Nevertheless, the use of monoclonal antibodies as the capture ligands may not optimize the capability for sensitive, rapid and reproducible detection of progesterone. Most of the studies presented utilized an inhibition immunoassay, which requires additional preparation and use of larger amounts of antibodies prior to analysis; a direct detection platform eliminates this additional step. Moreover, most of the detection strategies relied on modifying progesterone with linkers for immobilization to the sensor surface due to the small size of progesterone and its hydrophobic properties. This conjugation strategy can possibly alter the three-dimensional structure of the hormone and cause steric hindrance, thereby decreasing the sensitivity and reproducibility of the analytical measurements.

In this work, we introduce the use of progesterone X-aptamers as an alternative to monoclonal antibodies used in immunoassays for the ultrasensitive detection of progesterone in buffer. The setup that we adopt eliminates the need to covalently immobilize progesterone to the solid metal surface and preserves the activity and steric accessibility of progesterone.

We also aimed to enhance the small molecule sensing by using a SPRi nanoEnhanced sandwich assay to allow for ultrasensitive detection of progesterone. Aptamers are short single stranded oligonucleotide sequences, which mimic antibody binding to an analyte such as progesterone but with an enhanced sensitivity, specificity, and affinity^15^. Furthermore, the use of aptamers may circumvent the problems of steric hindrance that occur between small molecules and large antibodies and which can subsequently decrease the sensitivity of the detection platform^16^,^17^,^18^.

## Results

### Screening of developed X-aptamers

**Figure 2:**
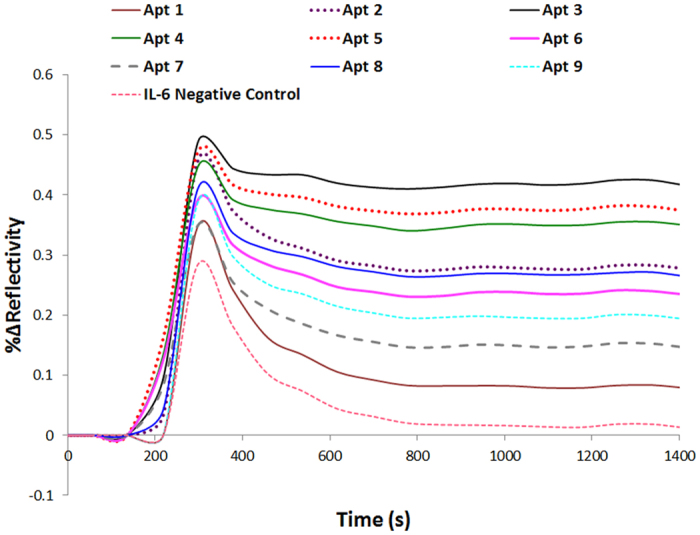
Screening of all X-aptamers using SPRi-based direct sensing. The SPRi sensorgrams of the interaction between progesterone (150 μM) and the 9 X-Aptamers developed and the negative IL-6 control aptamer spotted at 100 μM concentrations.

The biosensing assay was designed to achieve high specificity and selectivity for the detection of progesterone titers down to 1.575 ng/mL levels. Specific aptamers were selected and employed as the surface ligands. Taking advantage of the strong bond between biotin and avidin, the aptamers were tagged with a biotin molecule; an avidin surface was also employed for the direct attachment of the capture aptamer ligands to the sensing surface in a well-oriented manner. PEG800 was microwaved on the surface prior to the ligand immobilization since microwave irradiation of PEG-SH has been shown by a previous study to improve the effectiveness of the blocking process resulting in an enhanced SPRi response^19^. BSA (1%) and PEG2000 (1 mM) were further added to the surface inside the instrument to further block the sensing platform from non-specific adsorption of analyte. Long chain thiolated PEG offer the hydrophilic behavior that repels analyte molecules from binding non-specifically to the surface. The aptamers developed were screened for the detection of progesterone in the buffer (4% acetonitrile, 10 mM PBS) used to solubilize progesterone. Progesterone is highly hydrophobic. Therefore, acetonitrile was utilized to solubilize the steroid and enable its detection in a water-based buffer (10 mM PBS). The performance of each aptamer is presented in Fig. 2 in which the 9 aptamers evoked different SPRi signals after the injection of 50 μg/mL (150 μM) of progesterone. As shown, X-aptamers 3, 5 and 4 resulted in the highest SPRi response (%ΔR = 0.4 ± 0.01) upon the interaction with progesterone (150 μM); meanwhile, other X-aptamers experienced a lower binding response especially X-aptamer 1 which showed a similar SPRi signal to the IL-6 negative control. Furthermore, the kinetic profile of the three X-aptamers was evaluated using the SPRi ScrubberGen software, which cleans biosensor data and derives kinetic constants using a simple 1:1 interaction-fitting model. The dissociation constant (K_D_) was determined to be 21.74, 28.10 and 25.58 pM for X-aptamers 3, 4 and 5 respectively (Table 1). The DNA sequence and the base modifications performed on all X-aptamers are presented in Table 1.

### Direct Detection assay measurements

**Figure 3:**
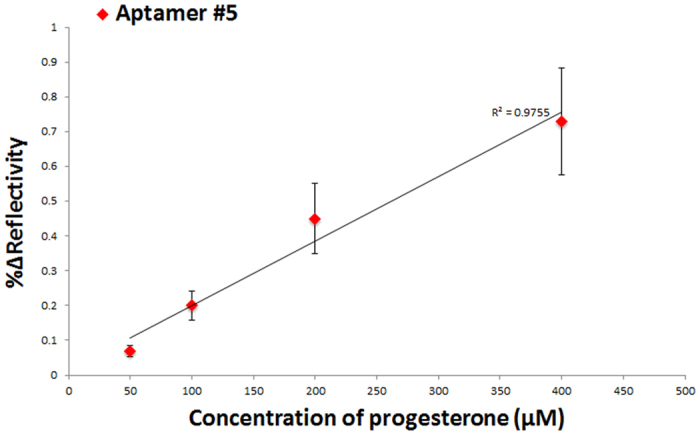
A direct detection concentration profile of SPRi response of progesterone in buffer. The SPRi signal is obtained from the interaction of X-aptamer #5 with progesterone in buffer at a concentration range (50–400 μM).

With the biosensing assay we were able to select several high binding activity aptamers from the library of aptamers developed that detected progesterone at a concentration of 49 μg/mL (150 μM). The next step was to test a range of concentrations (15.75, 31.5, 63, and 126 μg/mL) of progesterone and monitor the SPRi response of the binding to one of the top aptamers (Aptamer #5), which produced the largest SPRi response (%ΔR) upon interaction with progesterone. Aptamer 5 was spotted on the surface (N = 6 spots) along with the IL-6 aptamer (N = 6 spots) as a negative control; the kinetic analysis was performed. A linear correlation was obtained by increasing the concentration of progesterone injected to the functionalized surface and the results are presented in Fig. 3. As it can be shown, the limit of detection was concluded to be 15.75 μg/mL (50 μM) resulting in a response of %ΔR = 0.07 ± 0.01 for the direct biosensing assay.

### Specificity assay measurements

**Figure 4:**
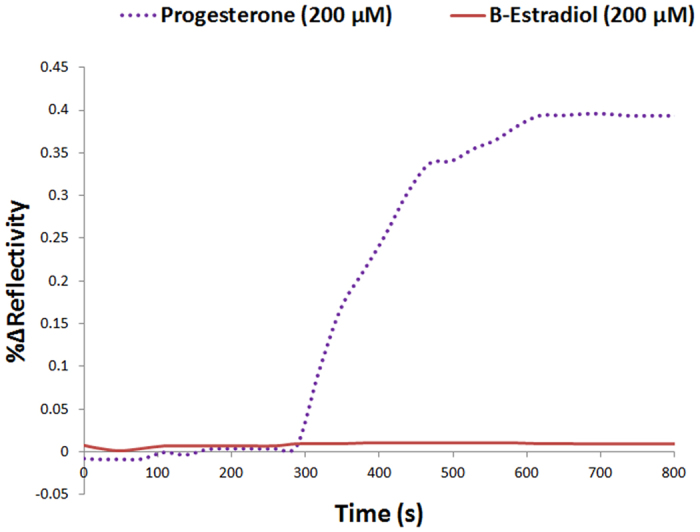
A specificity assays comparing the SPRi response of positive X-aptamer 5 to progesterone and ß-estradiol. SPRi sensorgrams of the response obtained from the interaction of aptamer #5 with progesterone (purple dotted line) and ß-estradiol (red solid line). Aptamer #5 demonstrated high-specificity as the SPRi signal obtained from the interaction with progesterone (63 μg/mL) is %ΔR = 0.40 ± 0.10 compared to a negligible signal after exposure to ß-estradiol (63 μg/mL).

Aptamers are well known for their enhanced selectivity and specificity as minor structural differences between biomolecules can be very well distinguished through the recognition process^20^. Taking this potential property into account, we examined the specificity of the positive aptamer (X-aptamer 5) by testing its detection against a closely related steroid, ß-estradiol (63 μg/mL) that coexists with progesterone in blood serum during the mid-menstrual cycle. The positive aptamer was chosen for further analysis due to its high binding SPRi signal shown in Fig. 2. The specificity assay was performed under the same conditions as the direct detection and utilized X-aptamer #5 with a solution containing ß-estradiol (63 μg/mL). As presented in Fig. 4, X-aptamer 5 showed significant specificity for only progesterone (%ΔR = 0.40 ± 0.10) and showed negligible binding to ß-estradiol.

### Sandwich-amplification assay

**Figure 5:**
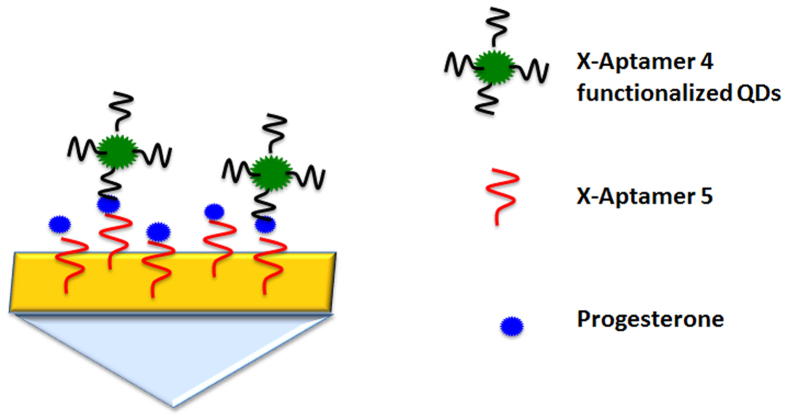
A schematic illustration of the signal amplification process of progesterone. The SPRi sensor surface is functionalized with X-aptamer #5. The first injection involves the introduction of progesterone and this is followed by the injection of X-aptamer #4 conjugated to the nanoEnhancers (NIR quantum dots).

**Figure 6:**
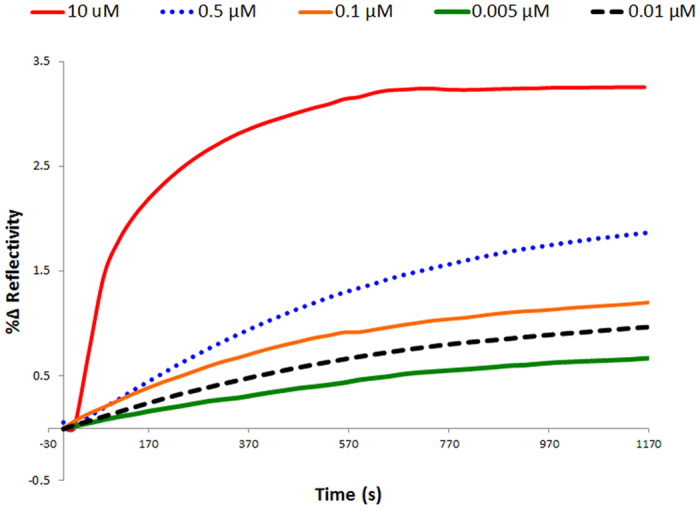
Normalized sensorgrams generated from the sandwich amplification assay. The curves represent the amplified signal from the interaction of the X-aptamer 5-progesterone with X-aptamer 4 conjugated to QDs at different concentrations of progesterone.

**Figure 7:**
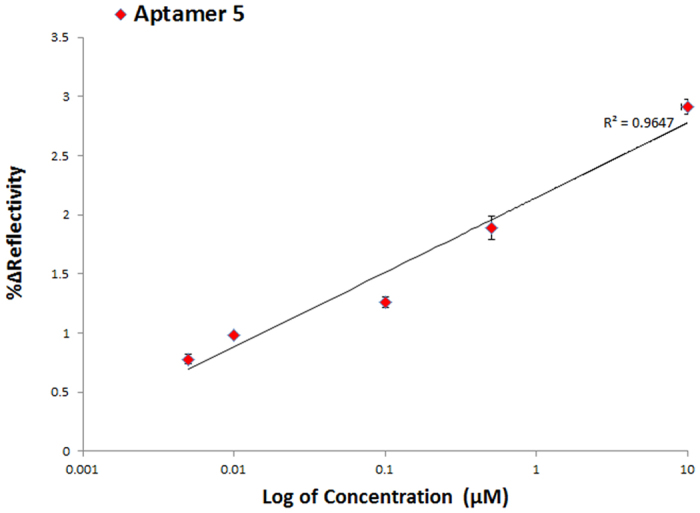
A sandwich-amplification assay concentration profile of progesterone in buffer. The SPRi response (%Δ Reflectivity) represents the interaction of nanoEnhancers with the various amount of progesterone (0.005–10 μM) upon binding to X-aptamer #5.

On the diagnostic front, SPRi assays could have tremendous impact if the capability to perform small molecule sensing at ultra-low concentrations is successfully achieved. As previously mentioned, small molecule sensing is still in its early stage of development because of the problem posed by low molecular weight. In this work, we employed nanoEnhancers (NIR quantum dots)^19^,^21^ as special signal amplification probes to generate a detectable signal at very low concentrations of progesterone. The sandwich assay was carried out with a two-step procedure. First, progesterone was injected followed by injection of the detection aptamer (X-aptamer #4) conjugated to the NanoEnhancers as shown in the following scheme (Fig. 5). X-aptamer 4 was tested as a detection aptamer due to its high binding as shown in the screening assay (Fig. 2). Moreover, X-aptamer 3, which is one of the high binding, X-aptamers, empirically did not perform as well as X-aptamer 4 in the sandwich pair format. X-aptamers 4 and 5 do not have any sequence similarity in the loop region, which could mean that they both target different binding regions of the analyte. However, X-aptamers 3 and 5 have a stretch of sequence similarity in the 5′ end of the loop region. The nanoEnhancers were added to further highlight the presence of progesterone. A range of concentrations was examined in this setup to determine the lowest detection limit and thus, evaluate the sensitivity of the biosensing assay. The SPRi sensorgrams of the tested concentrations in the sandwich assay are presented in Fig. 6. The limit of detection was determined to be 1.575 ng/mL (5 nM). The concentration profile of the tested concentrations of progesterone is represented in Fig. 7.

## Discussion

We developed specific X-aptamers for progesterone because this approach offers an advantage over conventional aptamers as well as monoclonal antibodies in the selective and ultrasensitive detection of progesterone. X-aptamers are small single stranded DNA molecules that contain nucleotides with amino acid functional groups and other small molecules attached. These chemical modifications confer vast chemical diversity to the X-aptamers, allowing for increased specificity when binding to targets. This chemical diversity is made possible by the micro bead based and non-PCR dependent selection process. In small molecule sensing, the accurate identification and detection of analyte necessitates highly sensitive capture ligands capable of efficiently binding to the analyte in solution in order to maximize the platform signal. Moreover, it requires that the capture ligands can discriminate between these structurally similar biomolecules that are present in the same complex samples. Therefore, introducing progesterone X-aptamers in combination with the NanoEnhanced biosensing capabilities of SPRi offer many advantages over currently used SPR-based and non-SPR based immunosensing assays.

A validation of the binding capability of the X-aptamers was performed using the SPRi based assay. The screening of all X-aptamers was performed and X-aptamers (3, 4 & 5) showed the highest SPRi response (%Δ Reflectivity) results. The SPRi response reflects the binding activity occurring on the surface; hence, the higher the response the higher the binding occurring between the analyte and immobilized ligand. For this reason, X-aptamer #5 was chosen for the direct detection as well as the sandwich-amplification sensing assay.

The surface setup is the first SPRi setup to our knowledge to detect progesterone in a mobile phase through the interaction with specific aptamer ligands bound to the sensor surface. Meanwhile, other SPR-based studies achieved the measurement in an inhibition assay in which progesterone was covalently bound to the metal surface and mobile antibodies were flown over the surface^12^,^14^. The presented sensor surface design consisted of immobilizing the progesterone X-aptamers on the surface. These progesterone aptamers were functionalized with a biotin reporter molecule at the 5′ end of the oligonucleotide since this would allow the aptamers to be immobilized on the sensing surface. The implemented modification in this assay offers the advantage of carrying direct measurements of progesterone offering practicality in the detection procedure. It also eliminates any alterations to the structure of progesterone mostly resulting from the different covalent modifications required to attach the steroid to the sensor surface. Furthermore, the mobile phase was optimized to allow complete solubilizing of progesterone, which is inherently insoluble in phosphate buffer. We accomplished that step by using a mixture of acetonitrile (4%) and phosphate buffer (10 mM) as the preparation buffer of samples and running buffer.

The use of amplification probes was designated to reach lower limits of detection and it significantly improved the performance of the biosensing assay. A detection limit of 1.575 ng/mL (5 nM) may be deduced from the range of low concentrations tested. The nanoEnhancers responsible for the improved sensitivity in the platform are made of heavy metal nanoparticles (quantum dots) that result in a high SPRi response once added to the bound detection probes. Moreover, the nanoEnhancers could display an energy coupling phenomenon with the oscillating surface plasmons on the metal surface as proposed by Malic *et al.*^21^. Therefore, both the mass loading effect and the energy-coupling phenomenon could amplify the effect the nanoEnhancers are demonstrating to the SPRi signal measured.

Label-free small molecule biosensing is a key area in the development of advanced diagnostic tools and analysis assays that can shed light on numerous biochemical processes. In this study, we developed a biosensing surface capable of performing analysis in a label-free and real time manner while demonstrating the ability to ultra sensitively and selectively distinguish between similarly structured hormones. We anticipate that further investigation to the platform design can extend the diagnostic application to detect a variety of different steroids in complex matrices such as serum and saliva. Moreover, several related steroids can be detected simultaneously by employing a multiplex assembled sensor surface immobilized with highly specific X-aptamers. This capability for the platform could prove to be particularly important because progesterone is a precursor in a complex steroid genic pathway that includes mineral corticoids, glucocorticoids, testosterone and estradiol.

## Methods

### Chemicals and materials

Bare gold chips were purchased from HORIBA Scientific (France). Nanostrip was purchased from Cyantek (CA, USA). 11-mercaptoundecanoic acid, N-Hydroxysuccinimide, Poly (ethylene glycol) methyl ether thiol (Mw = 800) and Poly (ethylene glycol) methyl ether thiol (Mw = 2000), extravidin, glycerol, ß-estradiol and acetonitrile were all purchased from Sigma Aldrich (PA, USA). 1-(3-Dimethylaminopropyl)-3-ethylcarbodiimide Hydrochloride was purchased from TCI America (PA, USA). Phosphate Buffered Saline, sodium chloride, and bovine Albumin Serum were purchased from Fisher Scientific (PA, USA). Progesterone was purchased from Cayman Chemical Company (MI, USA). Progesterone X-aptamers were selected using the AM Biotechnologies X-Aptamer kit and were synthesized by AM Biotech. Biotin-labeled interleukin 6 (IL-6) aptamers were purchased from base pair biotechnologies (TX, USA). Near-infrared streptavidin coated quantum dots were purchased from life technologies (CA, USA).

### X-aptamer selection

A library of X-aptamers bound to synthetic micro beads was provided by AM Biotech. Aptamer selection was carried out according to the protocol provided by AM Biotech. In addition to the negative selection suggested in the protocol (removal from the library of the aptamers that bind to streptavidin coated magnetic beads), a second negative selection was carried out to remove aptamers that bind to biotin. Streptavidin coated magnetic beads were mixed with the aptamer library, incubated for an hour and then were removed (along with bound aptamers) using a magnetic stand. The remaining library was incubated with 100 μM biotin for an hour and the biotin along with bound aptamers was pulled down through binding to streptavidin-coated beads. From the remainder of the aptamers in the library, primary selection of aptamers specifically binding to progesterone was performed by incubation of the library with biotinylated progesterone, followed by harvesting of the biotinylated progesterone along with bound aptamers using streptavidin coated magnetic beads. These harvested aptamers were cleaved from the synthetic micro beads using 1N NaOH. This pool of selected aptamers was divided into three aliquots. One aliquot was incubated with streptavidin coated magnetic particles. Aptamers bound to these beads were pulled down along with the magnetic particles. This fraction (fraction #1) represents aptamers that bind to streptavidin and magnetic beads. Second aliquot was incubated with biotinylated progesterone. The bound aptamers were pulled down along with biotinylated progesterone through binding to streptavidin-coated beads. This is the fraction containing progesterone-binding aptamers (fraction #2). The third fraction was used as a representative pool of all aptamers selected in the primary selection. All three fractions were PCR amplified with specific reverse primer for each fraction. The PCR products were combined and used as template for next generation sequencing. From the sequences identified, those that were enriched in the fraction #2 were chosen as the sequences specific to progesterone binding aptamers. Finally, AM Biotech using 9 of these selected sequences synthesized X-aptamers.

### Sensor chip functionalization

A bare gold sensor chip was cleaned under sonication using a nanostrip solution (50 °C, 90 mins) followed by thorough rinsing with ultrapure water. Then the sensor chip was placed in the UV/Ozone for 10 mins prior to surface functionalization. The first functionalization step with a layer of 11-MUA (34 mM in pure ethanol) was facilitated by microwave irradiation using a CEM discover microwave (50 W, 50 °C, 5 mins). The carboxyl groups of 11-MUA were activated by EDC first (65 mM in ultrapure water) in the microwave (50 W, 50 °C, 5 mins) and this was followed by reacting the surface with NHS (40 mM in ultrapure water) under the same conditions. Finally, PEG800 (1.25 mM in ultrapure water) is reacted with the sensor chip under the same microwave conditions. It is important to note that the sensor chip was washed thoroughly with ultrapure water between each functionalization step to ensure removal of unreacted chemicals. The chip was then dried with a stream of nitrogen and allowed to react with extravidin (0.2 mg/mL, 10 mM PBS) at room temperature and a relative humidity of 75% for 1 hour. After this step, the avidin chip was prepared for the ligand immobilization step by a thorough rinse with ultrapure water and dried with a stream of nitrogen.

### Ligand immobilization

The avidin sensor chip was spotted with the biotin-labeled library of progesterone X-aptamers and negative interleukin 6 aptamer. In the direct detection-sensing assay, the spotting concentration of both positive progesterone and the negative control IL-6 aptamers was 100 μM and prepared in a phosphate spotting buffer (10 mM PBS, 10% glycerol, PH = 7.4) solution. In the sandwich amplification-sensing assay, the optimal spotting concentration of both positive progesterone and negative control IL-6 aptamers was 5 μM and prepared in phosphate spotting buffer (10 mM PBS, 10% glycerol, PH = 7.4). The spotting was performed using SPRi-Arrayer and 300 μm Teflon spotting pin. The biotin-labeled X-aptamers were allowed to react with the avidin surface at room temperature and a relative humidity of 75% for 2 hours.

### SPRi measurements

The SPRi measurements were performed in a temperature-controlled HORIBA Scientific SPRi-PLEX II apparatus. The sensing mechanism utilized an incident monochromatic light that hits the sensor surface, a detector that collects the resulting reflected light and a CCD camera that generates a real-time contrast image. The sensor surface consists of a high index prism, coated with and a gold layer (50 nm) where the ligands of interest are immobilized. At a specific incident angle known as the resonance angle, the impinging light excites the free electrons in the gold layer; resulting in an evanescent field (up to ~400 nm); which exponentially decays out from the surface. And this in turn leads to a dip in the reflected light. The surface plasmons thus created are sensitive to perturbations in the refractive index of the surrounding medium; and a detector measures these alterations as changes in the reflectivity signal. The SPRi apparatus also allows a real-time visualization of the sensing surface for multi-array purposes by intercepting the reflected light with a CCD camera to provide a contrast image reflecting biomolecular interactions at the surface.

### NanoEnhanced binding assay

The sensor surface was inserted into the SPRi instrument for assay analysis and a phosphate buffer (10 mM PBS, 4% Acetonitrile, PH = 7.4) buffer was degassed and allowed to flow above the surface at room temperature and a flow rate of 5 μL/min. Further blocking of the sensing surface was performed inside the instrument by sequentially injecting a BSA solution (1%) followed by a PEG2000 solution (1 mM) and the non-bound molecules were washed properly from the surface between steps. A calibration step using a solution of sucrose (3 mg/mL, 10 mM PBS PH = 7.4, 4% Acetonitrile) was then performed and that precedes the injection of the analyte. The samples injected were diluted in the running buffer and loaded to a 150 μL injection loop. In the direct detection-sensing assay, a solution of progesterone prepared in a 10 mM PBS and 4% acetonitrile solution was injected and the kinetics of the binding event were monitored. And in the sandwich amplification-sensing assay, the primary injection consisted of a specific concentration of progesterone sample followed 5 minutes after by the second injection consisting of a progesterone detection aptamer (X-aptamer #4) conjugated to the quantum dots. The signal amplification sample consisting of the detection aptamer (20 nM) was allowed to incubate with the streptavidin conjugated quantum dots (10 nM) for 30 mins prior to injection above the sensing surface. And the kinetics of both steps was monitored in real-time. Finally, the SPRi sensorgrams were analyzed and the final plots were prepared using the ScrubberGen horiba software.

## Additional Information

**How to cite this article**: Zeidan, E. *et al.* Nano-SPRi Aptasensor for the Detection of Progesterone in Buffer. *Sci. Rep.* **6**, 26714; doi: 10.1038/srep26714 (2016).

## Figures and Tables

**Figure 1:**
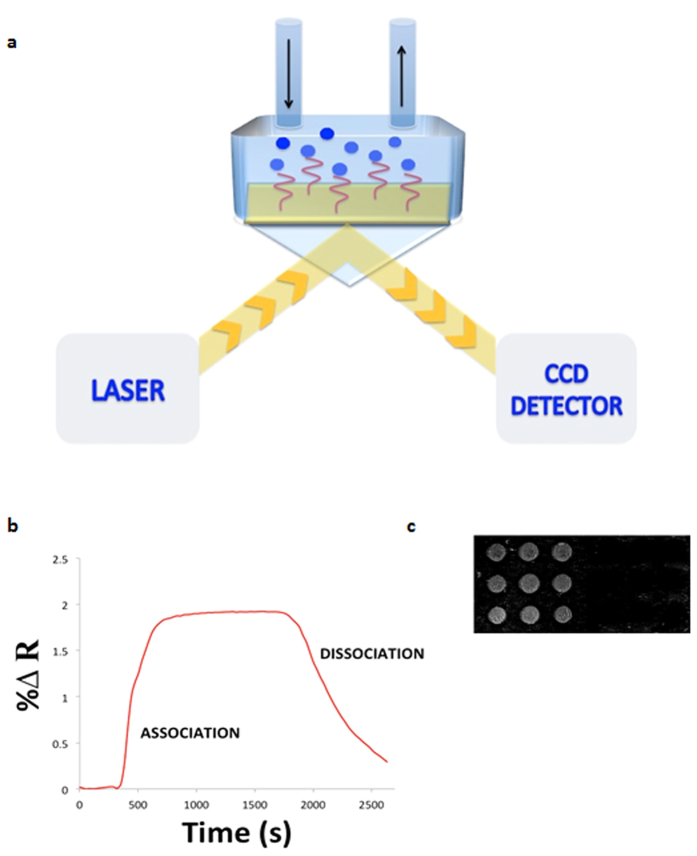
The SPRi biosensor setup based on the Kretschmann geometry. (**a**) The light incident on the metal surface excites the surface plasmons (SPs) causing a dip in the reflected light. A CCD detector collects the reflected light and measures changes in reflectance. (**b**) The detector generates an association and dissociation sensorgram reflecting the biomolecular interactions on the surface. (**c**) A real-time digital difference image is generated to reflect the biomolecular binding (bright spots) versus no binding (dark spots).

**Table 1:** Kinetic analysis of dissociation constant of all X-aptamers using ScrubberGen SPRi analysis software.

X-Aptamer	K_D_ (pM)	SD	DNA Sequence
1	999.74	0.069	TTTTT TT GATC CTTC--TCGTAYTGTCCACTGCGATCWCTT GATC TTTTT
2	49.51	0.099	TTTTT TT CTAG CTTCGTYTGTCTCAACGTGCCCTGCGWCGT GATC TTTTT
3	21.74	0.087	TTTTT TT CTAG AGATGTYTGTCTCAACGTGCCCTGCGWCGT GATC TTTTT
4	28.10	0.107	TTTTT TT CTAG AGAAGTYTGTCTTTCGCAYCAAGAGWTACG GATC TTTTT
5	25.58	0.081	TTTTT TT CTAG CTTCCGTGAAAYCAACGTGCCCTGGWWCGT CTAG TTTTT
6	53.03	0.061	TTTTT TT CTAG CTTCCGTGAAAYCAACGTGCCCTGGWTAGT CTAG TTTTT
7	140.37	0.064	TTTTT TT CTAG CTTCCGTGAAAYCAACGTGACCTGWWTAGT GATC TTTTT
8	40.79	0.076	TTTTT TT CTAG CTTCCGTGAAAYCAACGTGCCCTGGWAWCW GATC TTTTT
9	61.86	0.076	TTTTT TT CTAG CTTCCGTGAAAYCAACGTGCCCTGWWAWCG CTAG TTTTT

The dissociation constant (KD value) reported was calculated for all 9 X-aptamers along with the standard deviation value (SD).

W = dU with indole (tryptophan) attached to position 5.

Y = dU with phenol (tyrosine) attached to position 5.
